# Unraveling the Nanosecond Photoresponse of Layered HgPSe_3_

**DOI:** 10.1021/acs.nanolett.4c04945

**Published:** 2025-02-11

**Authors:** Nikolas Antonatos, Artur P. Herman, Beatriz de Simoni, Karolina Ciesiołkiewicz, Eduard Belas, Marián Betušiak, Roman Grill, Kalyan Jyoti Sarkar, Amutha Subramani, David Sedmidubský, Valentino Jadriško, Alessandro Baserga, Micol Bertolotti, Stefano Dal Conte, Christoph Gadermaier, Giulio Cerullo, Antonella Treglia, Annamaria Petrozza, Robert Kudrawiec, Zdeněk Sofer

**Affiliations:** † Department of Inorganic Chemistry, University of Chemistry and Technology Prague, Technická 5, 166 28 Prague 6, Czech Republic; ‡ Department of Semiconductor Materials Engineering, Wrocław University of Science and Technology, Wybrzeże Wyspiańskiego 27, 50-370 Wrocław, Poland; § Faculty of Mathematics and Physics, Institute of Physics, Charles University, Ke Karlovu 3, 121 16 Prague 2, Czech Republic; ∥ Department of Physics, Politecnico di Milano, Piazza Leonardo da Vinci 32, I-20133 Milano, Italy; ⊥ Center for Nano Science and Technology @PoliMi, Istituto Italiano di Tecnologia, Via Rubattino 81, 20134 Milan, Italy

**Keywords:** mercury selenophosphate (HgPSe_3_), fast response photodetector, layered material, metal phosphorus trichalcogenides, transient reflectivity, pump−probe spectroscopy

## Abstract

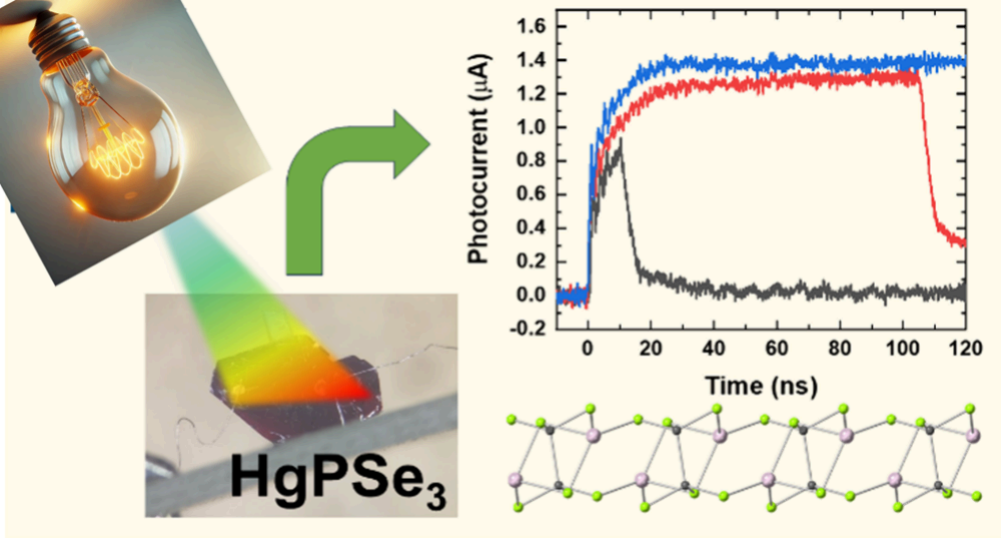

Chalcogen phosphates of transition metals make up a well-known group of antiferromagnetic semiconductors with the general formula MPX_3_, where M represents a transition metal and X is a chalcogen, either sulfur or selenium. Most of these compounds adopt a similar structure; however, mercury phosphochalcogenides present an exception with their unique van der Waals layered structure. Transition metal chalcogenides are highly appealing materials for photodetectors due to their exceptional optoelectronic properties. Among them, HgPSe_3_, a layered van der Waals phosphoselenide, shows promise for photodetection over a broad spectral range, from visible light to X-rays. Despite this, the electronic processes governing its photoresponse remain unclear. In this study, we demonstrate a nanosecond response time of a HgPSe_3_-based photodetector to visible light and gain deeper insights into the underlying charge carrier dynamics through a comprehensive investigation using complementary time-resolved experimental techniques. Our findings on the role of carrier traps provide a potential pathway for optimizing optoelectronic device performance.

Photodetectors are essential devices that possess the ability to convert optical signals directly into electrical signals, finding applications in a diverse range of fields.^1−5^ Transition metal chalcogenides (TMCs) have been extensively investigated,^6^ mainly in the domain of field-effect transistors (FETs),^7−9^ solar cells,^10,11^ light-emitting diodes (LEDs),^12,13^ and photocatalysis.^14^ In recent years, there has been a shift in focus toward emerging TMC photodetectors, driven by their high mobility–lifetime product, long carrier diffusion length, cost-effectiveness, and simple fabrication processes. This area of research holds great promise for expanding the potential applications of TMC materials beyond FETs.^15,16^

A new and emerging family of layered van der Waals TMCs, based on metal phosphorus trichalcogenides (MPX_3_), has attracted a significant amount of attention due to their unique optoelectronic properties.^17^ These are layered materials in which each layer consists of a structure of metal and phosphorus atom sheets sandwiched between two sheets of chalcogen (S or Se) atoms held together by ionic bonds. The choice of chalcogen X and the broad selection of metals M enable the tuning of the bandgap and the photoresponse over an outstandingly broad spectral range.^18^ The appealing properties have spurred investigations into their potential applications in light-harvesting systems, such as the hydrogen evolution reaction (HER),^18−21^ water-splitting reactions,^22−24^ and photodetectors.^25−27^

HgPSe_3_ is an intriguing member of the MPX_3_ family that has garnered attention due to its unique structural, optical, electronic, and magnetic properties.^28,29^ HgPSe_3_ exhibits a crystal structure composed of interconnected octahedra, with mercury (Hg) ions surrounded by phosphorus (P) and selenium (Se) atoms illustrated in Scheme S1 and the crystallographic parameters listed in Table S1. This material stands out due to its bandgap of ∼2 eV and excellent light absorption properties, demonstrating an outstanding photoresponse in the range from X-ray to visible wavelengths with high sensitivity (88.82 μC Gy^–1^ cm^–2^ under X-ray illumination).^30^ Therefore, HgPSe_3_ has been demonstrated to be an excellent broadband photodetector. Its low-cost synthesis and simple fabrication process add to its appeal for practical applications. While research on HgPSe_3_ is still scarce, these initial findings open up exciting opportunities for exploring its potential in areas such as photodetection, photovoltaics, and other optoelectronic devices. The knowledge of photocarrier dynamics is essential for the application of semiconductors in optoelectronic devices. Herein, combining a number of time-resolved techniques, such as differential optical reflectance spectroscopy, photoluminescence, microwave conductivity, and photocurrent measurements, we unravel the mechanisms that enable ultrafast photodetection in HgPSe_3_ with a response time on the order of 10 ns. Our results provide fundamental information for utilizing this material as a photodetector as well as in other optoelectronic devices.

HgPSe_3_ was prepared following the synthetic route described in detail in ref (30) and in the Supporting Information. The high quality of the resulting layered crystals is confirmed via X-ray diffraction, as well as Raman spectroscopy, energy dispersive spectroscopy (EDS), and X-ray photoelectron spectroscopy (XPS) in Figures S2–S4, respectively, and is discussed in detail in the Supporting Information. The scanning electron microscopy (SEM) image in Figure 1a illustrates the layered nature of the material. The device used for photocurrent measurements is shown in Figure 1b, and a diagram of this device is provided in Scheme S2. It consists of a HgPSe_3_ crystallite glued to the sample holder with toluene-based glue and a thin alumina support on the back of the sample. The sample is equipped with Au intergrid contacts with a 0.3 mm contact pitch. The surface of the sample was quite uneven, and thus, parts of the gold contacts had to be connected with silver paint to ensure conductive connections.

**Figure 1:**
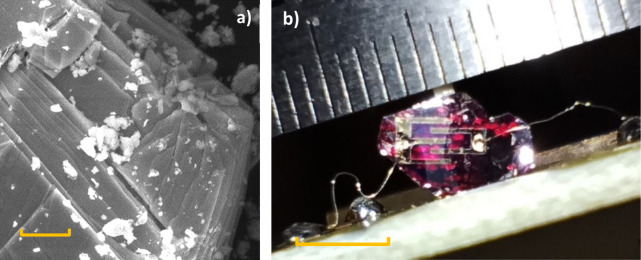
(a) SEM image of bulk HgPSe_3_. The scale bar is 5 μm. (b) Image of the HgPSe_3_ thin film used for photocurrent measurements with the Au contacts. The scale bar is 5 mm. A schematic diagram of the sample is shown in Scheme S2.

The photocurrent response of HgPSe_3_ in Figure 2 almost perfectly follows the rectangular optical pulse, particularly for pulse durations of ≥100 ns. In Figure S5, the linear parts of the rising and falling edges correspond to the rise (τ_r_) and fall (τ_f_) times, respectively, of the optical power transient of the laser diode recorded with a commercial photodetector with a subnanosecond response time. The photocurrent transients in Figure 2 follow the optical power transients with a high degree of precision, demonstrating a response time of significantly shorter than 9 ns, which is independent of the applied voltage. This independence from the electric field indicates that the photocurrent transient is not related to the carrier mobility, which would become faster with a higher field. Hall-effect measurements of the 257 μm HgPSe_3_ flake used in these experiments determined a carrier mobility of 63.08 ± 16.99 cm^2^ V^–1^ s^–1^. Additionally, the trap density was estimated to be on the order of 10^14^ cm^–3^, with calculation details provided in the Supporting Information. On the contrary, such a fast field-independent photocurrent transient is consistent with a population of photogenerated charges with a lifetime of <10 ns, which reaches an equilibrium between photogeneration and recombination/trapping on defects after several time constants. The tail following the optical pulse is more pronounced and decays more slowly for longer pulses, i.e., larger total number of absorbed photons per pulse. The characteristic tail decay times corresponding to the fall times (τ_f_) are 12, 23, and 37 ns for 10 ns, 100 ns, and 1 μs long optical pulses, respectively (Figure S5). This behavior is likely caused by the release of photogenerated electrons/holes trapped at defect levels, whose population builds up during the whole pulse duration and has a distribution of lifetimes, leading to a decay that depends on the population density of the defects and hence on the pulse duration.

**Figure 2:**
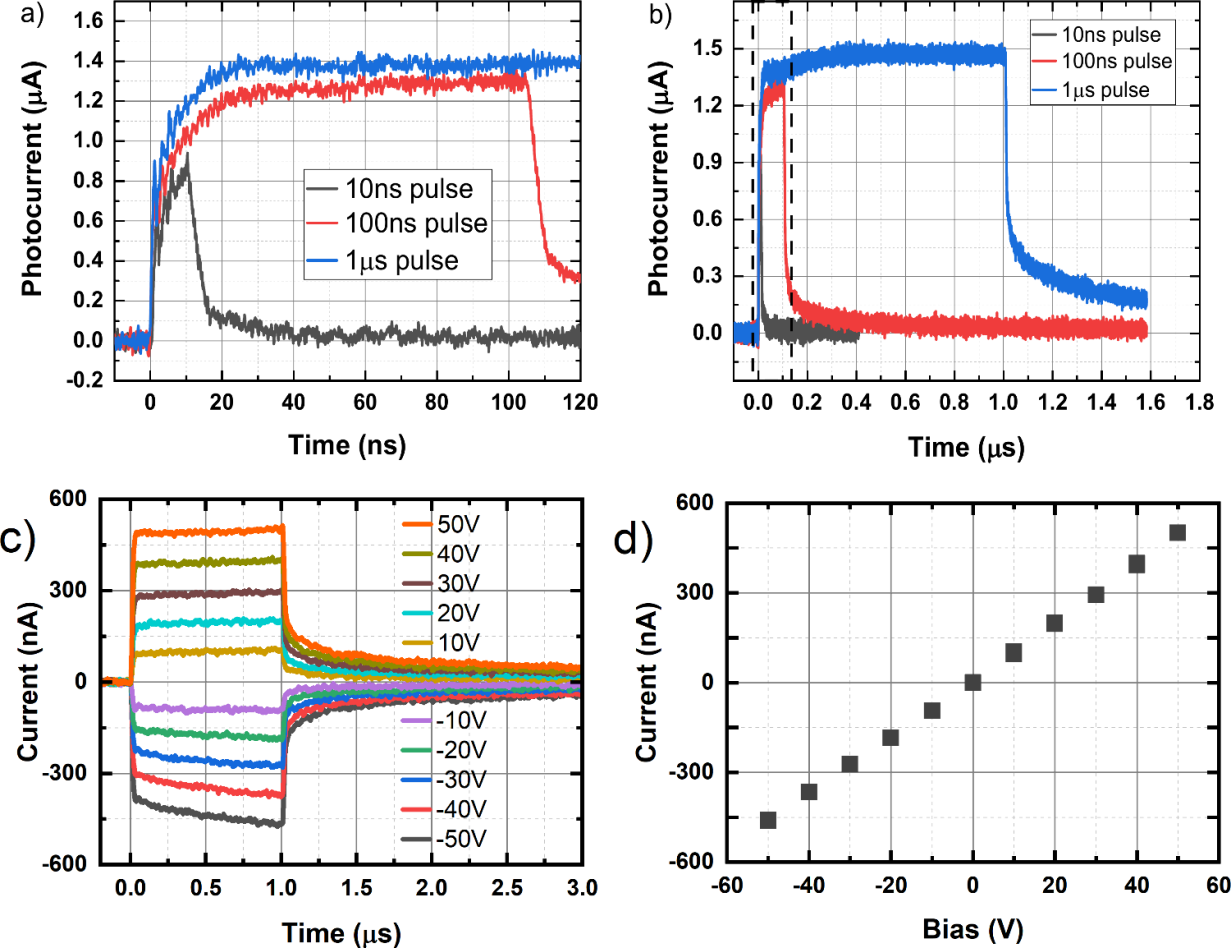
Dependence of the HgPSe_3_ photocurrent on the width of the optical pulse at 50 V bias. Panel a is an enlarged depiction of panel b in the 0–120 ns region, marked with a dashed frame. The current pulses were excited by optical pulses with a wavelength of 405 nm for 10 ns, 100 ns, and 1 μs durations, with a repetition time of 10 μs. (c) Photocurrent transients and (d) photocurrent bias voltage characteristics of HgPSe_3_. The sample is illuminated by a single optical pulse (wavelength of 405 nm, width of 1 μs, delay of 1 ms) per bias pulse (5 ms + 5 ms, 90 ms depolarization) (see Figure S1b for the optical pulse delay definition).

Additionally, Figure 2c shows the photocurrent transients for different bias voltages of the device. The photocurrent response is linear in the bias voltage without the typical saturation, described by the Hecht relation,^31^ where all photogenerated charge is collected.

The photocurrent transients simply scale linearly with the bias voltage without changing their shape, suggesting field-independent dynamics for the free and trapped carriers (Figure 2d). This result testifies to a dominating generation–recombination quasi-equilibrium at the photoexcitation where the loss of photocarriers at the contact does not affect the free carrier density. As shown in Figure 2c, the maximum photocurrent observed is ∼500 nA, corresponding to an excitation intensity that significantly exceeds 3 × 10^12^ photons s^–1^. This intensity corresponds to a power of ∼1 μW. This discrepancy further corroborates the strong recombination of photogenerated carriers and the linear dependence of the photocurrent on bias. Given the coplanar geometry of the device, an increased level of recombination of carriers is expected because the photogenerated electrons and holes do not separate as they would in planar–planar devices.

To relate the photocurrent response to the underlying carrier dynamics, we studied time-resolved microwave photoconductivity (TRMC) and time-resolved photoluminescence (TRPL). Because this compound has a direct bandgap,^30^ bandgap-related photoluminescence (PL) is observed at room temperature. Following the assignment of the emission and reflection features developed in ref (30), we analyze the temperature evolution of the PL band and its direct comparison with the reflectance (R) spectrum, in which the free exciton (FX) is observed (Figure 3a). It must be noted that free excitons can exhibit line broadening due to factors such as phonon coupling, disorder, or defects. When these broadening mechanisms are strong, they may obscure a clear peak by spreading the excitonic feature over a wider energy range, leading to a weaker and less distinct signal. At low temperatures, the near-band-edge emission is dominated by bound excitons (BX), which dissociate quite quickly with an increase in temperature. Above 60 K, the PL spectra are dominated by FX instead of BX (Figure 3a). As the temperature increases further, the FX emission shifts toward the red, and its intensity decreases significantly as a result of the dissociation process promoted by the increasing thermal energy. The temperature dependence of the intensity of the FX peak is shown in Figure 3b, and the exciton binding energy determined from this dependence is 72 ± 6 meV, which is quite high but typical for layered semiconductors in bulk form.^32^ This means that at room temperature we are dealing with both FX and band-to-band (b–b) recombination, because *kT* is approximately one-third of the exciton binding energy.

**Figure 3:**
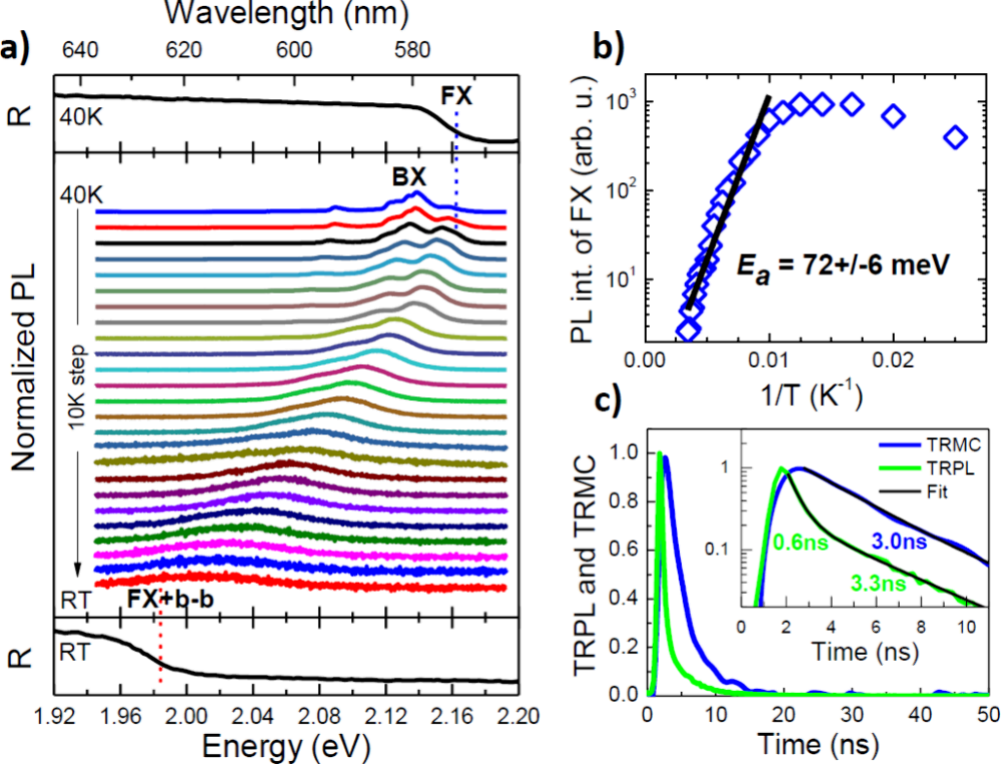
(a) Direct comparison of reflectance (R) and micro-PL spectra measured from 40 K (PL dominated by bound excitons, BX) to room temperature (RT) (PL spectra dominated by broad overlapping peaks from free exciton and band to band recombination, FX + b–b). (b) Analysis of the intensity of free exciton (FX) PL as a function of temperature. (c) Time-resolved microwave photoconductivity (TRMC) and time-resolved PL (TRPL) measurements at room temperature.

The PL decay curve measured for the emission peak at room temperature (2.0 eV) consists of two components with time constants of 0.6 ± 0.1 and 3.3 ± 0.2 ns, which we attribute to FX and b–b recombination, respectively. This is due to the spectral overlap of these emissions caused by their thermal broadening and to the broad spectral detection window in TRPL measurements, which is 50 meV. In the case of TRMC, excitons do not participate in current conduction (they do not generate the TRMC signal) because they are neutral particles,^33^ and therefore, the TRMC signal is assigned to free carriers in HgPSe_3_. The free carrier lifetime determined from the TRMC decay curve is 3.0 ± 0.2 ns and is very consistent with the longer component of the TRPL decay curve. Hence, we assign the shorter component of the TRPL decay to the FX emission. Interestingly, unlike in the case of other layered materials (TMDs)^33^ we did not observe long-lived carriers in TRMC measurements, suggesting a low concentration of deep trap states.

Compared to conventional direct-gap semiconductors, the observed decay times are quite long, which can be related to the high quality of the studied crystal and the nature of the direct bandgap, which is close to the quasi-direct gap,^30^ and thus favors the occupation of the adjacent conduction band minimum by carriers, especially at higher temperatures, including room temperature. The photodetector response time is comparable to those of both TRMC and TRPL decays, suggesting that it is limited by the carrier lifetime rather than by the transport properties of the material.

To gain deeper insight into the photocurrent dynamics, we measured the nanosecond transient optical reflectance change Δ*R*/*R* on a HgPSe_3_ crystal in a pump–probe configuration. The photoinduced change in reflectivity, shown in Figure 4, following photoexcitation with a 1 ns pulse at 2.33 eV, well above the bandgap, shows three main peaks, at the energy from the main reflectance resonance upward, pointing to a rich variety of direct interband transitions and/or exciton species accessible upon photoexcitation.

**Figure 4:**
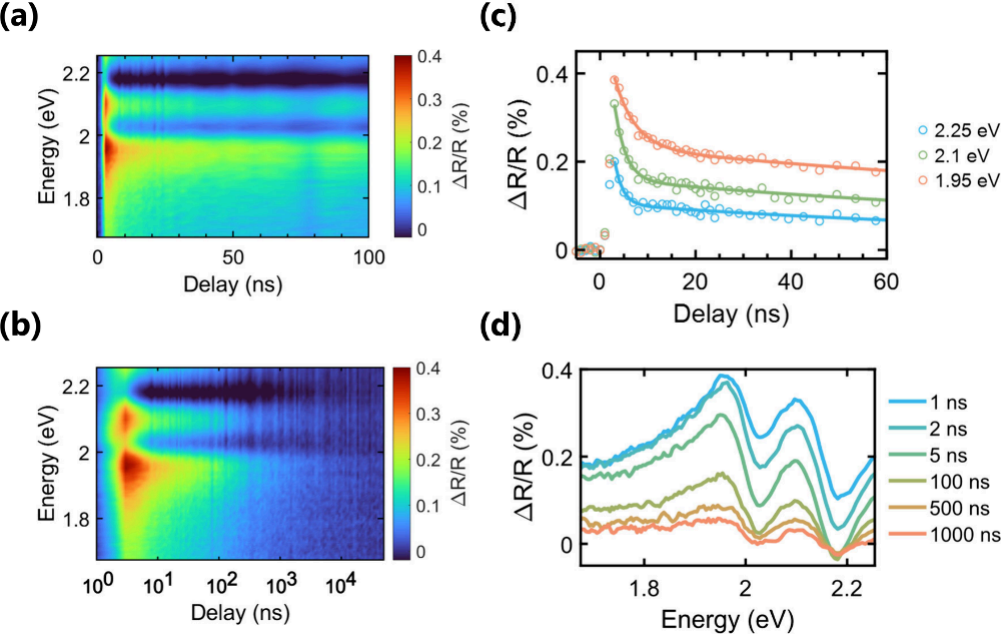
(a and b) Contour plots of transient differential reflectivity *ΔR*/*R* of a HgPSe_3_ crystal excited at 2.33 eV with a fluence of 300 μJ cm^–2^. (c) Temporal *ΔR*/*R* dynamics at selected probe energies: (○) experimental data and (—) biexponential fit with τ_1_ = 4 ps and τ_2_ = 140 ps. (d) Spectra at selected pump–probe delays.

Band structure calculations^30^ predict several bands below the valence and above the conduction band with spacings of 100–300 meV. Hence, the population of the band extrema can bleach several closely spaced transitions, resulting in the multiple peaks observed here. For increasing pump–probe delays, the transient signal decays without any major evolution in the spectral shape, suggesting that the signal originates from one major photoexcited state population, the carriers at the band extrema. The temporal evolution of this population is dominated by two exponential decays with time constants of 4 and 140 ns, followed by a non-exponential tail that continues into the microsecond range. The faster decay is very similar to the photocurrent response in Figure 2 as well as the TRMC and the slower TRPL decay. We therefore ascribe this component to the recombination of mobile charge carriers. The slower dynamics are similar to the tails in the photocurrent decay in Figure 2, which we ascribed to the release of trapped carriers. Similarly long lifetimes of trapped carriers have been observed in the transient absorption dynamics of other two-dimensional semiconductors such as lead halide perovskites^34^ and transition metal dichalcogenides.^35^ Therefore, we attribute the slower decay dynamics to the release and recombination of trapped carriers.

In conclusion, we have studied the nanosecond temporal dynamics of photogenerated carriers in HgPSe_3_ by using the complementary time-resolved photocurrent, microwave conductivity, photoluminescence, and differential reflection spectroscopy. We have demonstrated an ultrafast photodetector response in the order of 10 ns, governed by the recombination time of mobile photogenerated charges. Additionally, we have identified trapped charges with much longer lifetimes, whose slow release limits the recovery of the photodetector in proportion to the buildup of the trapped population. These results demonstrate the potential of this material and provide fundamental information for understanding its behavior in photodetectors and other optoelectronic devices. Further study of the carrier traps will lead to guidelines for performance optimization.

## Data Availability

The data sets generated and/or analyzed during the study are accessible via the Zenodo repository 10.5281/zenodo.13892435.

## Supporting Information Available

Crystal structure and crystallographic parameters of HgPSe_3_, detailed description of experimental methods, and structural and morphological analysis using XRD, Raman spectroscopy, SEM, EDS, and XPS (PDF)
