# Supplementary material for: Unraveling the Nanosecond Photoresponse of Layered HgPSe3
Source: Nano Lett. 2025 Feb 11;25(8):3053–8. doi: 10.1021/acs.nanolett.4c04945 (PMC11869261; doi:10.1021/acs.nanolett.4c04945)
Supplement: Supplementary file 1 — nl4c04945_si_001.pdf [file nl4c04945_si_001.pdf]

Supporting Information

Unraveling the Nanosecond Photoresponse of Layered $HgPSe_3$

Nikolas Antonatos[1,2], Artur Herman[2], Beatriz de Simoni[2], Karolina Ciesiołkiewicz[2], Eduard Belas[3], Marián Betušiak[3], Roman Grill[3], Kalyan Jyoti Sarkar[1], Amutha Subramani[1], David Sedmidubsky[1], Valentino Jadriško[4], Alessandro Baserga[4], Micol Bertolotti[4], Stefano Dal Conte[4], Christoph Gadermaier[4], Giulio Cerullo[4], Antonella Treglia[5], Annamaria Petrozza[5], Robert Kudrawiec[2], Zdeněk Sofer[1]*

[1]Department of Inorganic Chemistry, University of Chemistry and Technology Prague, Technická 5, 166 28 Prague 6, Czech Republic; zdenek.sofer@vscht.cz

[2]Department of Semiconductor Materials Engineering, Wrocław University of Science and Technology, Wybrzeże Wyspiańskiego 27, 50-370 Wrocław, Poland

[3]Faculty of Mathematics and Physics, Institute of Physics, Charles University, Ke Karlovu 3, 121 16 Prague 2, Czech Republic

[4]Department of Physics, Politecnico di Milano, Piazza Leonardo da Vinci 32, I-20133 Milano, Italy

[5]Center for Nano Science and Technology @PoliMi, Istituto Italiano di Tecnologia, Via Rubattino 81, 20134 Milan, Italy

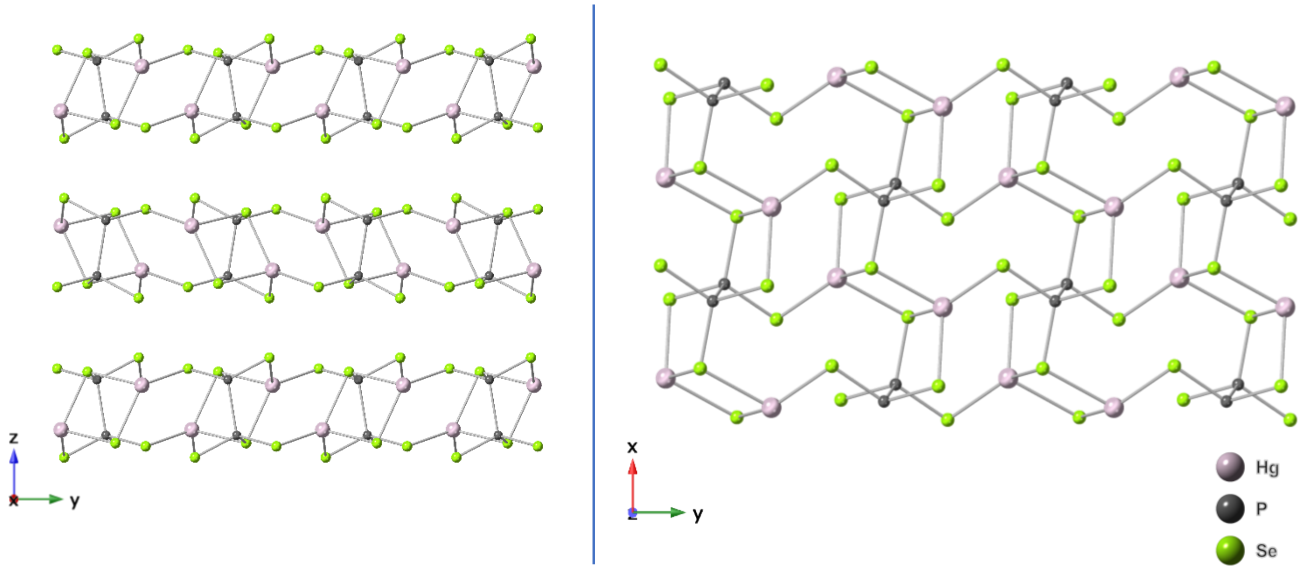

Scheme S1. Crystal structure of $HgPSe_3$. Side view (left) and top view (right).

Table S1. Crystallographic parameters of $HgPSe_3$.

**$HgPSe_3$ crystal structure parameters**	
**Crystal structure**	Monoclinic
**Space Group**	*C2/c*
**a**	6.55 Å
**b**	11.38 Å
**c**	13.61 Å
**α**	90.0°
**β**	98.47°
**γ**	90.0°
**Cell Volume**	1002.38 $Å^3$

1. Materials and Methods

Material synthesis

$HgPSe_3$ was synthesized by placing mercury (99.999%, Strem, Germany), red phosphorus (99.999%, Strem, Germany), and selenium (99.999%, Strem, Germany) in a quartz ampoule in stoichiometric quantities to a total amount of 15 g together with 0.25 g of HgI_2 acting as a transport agent. The ampoule was melt-sealed with an oxygen-hydrogen flame at a pressure of $1x10^{-3}$ Pa. Liquid nitrogen was employed to prevent mercury evaporation during the sealing process. The ampoule was then placed in a crucible furnace and heated at 400 °C while the cold end was kept below 200 °C for 48 h. Afterward, the ampoule was moved to a horizontal two-zone furnace where the growth zone was heated at 450 °C while the reaction mixture was kept at 350 °C for 24 h. Then, the gradient was reversed, and the changed zone was kept at 400 °C while the growth zone decreased from 350 to 300 °C in the course of 5 days. Finally, the ampoule was opened inside an argon glovebox.

Structural and Morphological Characterization

X-ray diffraction (XRD) patterns were acquired using a Bruker D8 Discoverer powder diffractometer (Bruker, Germany) in Bragg-Brentano parafocusing geometry and applying Cu K_α radiation (λ = 0.15418 nm, U = 40 kV, I = 40 mA). The diffraction patterns were collected between 5° and 90° of 2θ with a step size of 0.020° and the acquired data was evaluated using HighScore Plus 3.0e. The Raman spectrum was taken by an inVia Raman microscope (Renishaw, England) in backscattering geometry with a CCD detector and a DPSS laser (532 nm, 50 mW) with applied power of 5 mW and 20x magnification objective. A small amount of the bulk material was placed on a piece of silicon wafer and the morphology of bulk $HgPSe_3$ was investigated via scanning electron microscopy (SEM) images through a field emission gun

electron source (Tescan Lyra dual microscope) and elemental composition and mapping of the materials were obtained by an energy dispersive spectroscopy (EDS) analyzer (X-MaxN) with a 20 mm^2 SDD detector (Oxford Instruments) and AZtecEnergy software. The sample was placed on a carbon conductive tape. SEM and EDS measurements were carried out using an electron beam in the range of 5-10 kV. grid (Cu; 200 mesh; Formvar/carbon). X-ray photoelectron spectroscopy (XPS) measurements were performed in an ESCAProbeP spectrometer (Omicron Nanotechnology Ltd, Germany) employing a monochromatic aluminum X-ray radiation source (1486.7 eV). Wide-scan surveys of all elements were performed with subsequent high-resolution scans of mercury (Hg 4f), phosphorus (P 2p), and selenium (Se 3d). The samples were placed on a Si wafer. An electron gun (1-5 V) was utilized to eliminate the sample charging during measurement. All XPS survey spectra were afterward analyzed by CasaXPS software.

Time-resolved photocurrent relaxation

Photocurrent relaxations were measured under pulsed illumination and bias and recorded by a digital oscilloscope (LeCroy 610Zi). The setup for photocurrent measurement is shown in **Figure S1a**). Pulsed bipolar bias is supplied by the arbitrary waveform generator (Tektronix AFG31052) and fast bias amplifier (Falco WMA-300). The bias waveform displayed in **Figure S1b**) is used to suppress ionic migration (bipolar) and space charge formation (depolarization time >100ms; the sample is grounded). This is even more necessary in halogen-based perovskites such as $MAPbI_3$ [1]).

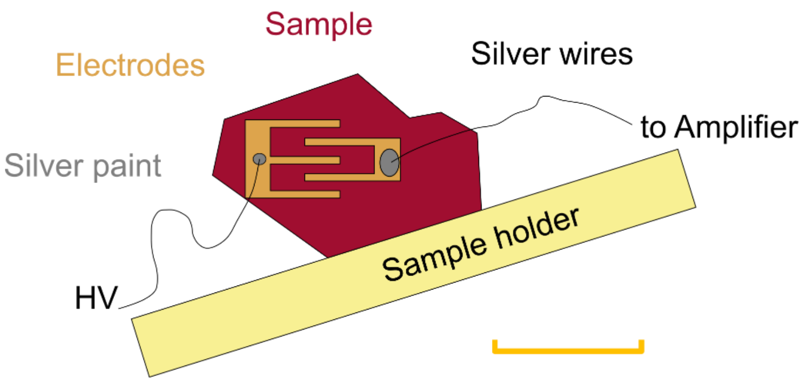

Scheme S2. Schematic diagram of the sample and the device depicted in **Figure 1b**. Scale bar is 5 mm.

A 405 nm (3.06 eV) laser diode is used to illuminate whole electrodes (diameter ~ 5 mm). The width (10 ns – 1 μs) and delay of the optical pulse are controlled by the arbitrary waveform generator. Because of the closely spaced interdigitated electrodes, it is impossible to distinguish between electron and hole photocurrent. The signal is then amplified and recorded by the oscilloscope. The AC coupling and amplifier distort the measured signal. An accurate undistorted signal can be obtained by deconvolution with the circuits transfer function. The sample was mounted on the edge (see **Figure 1b**) with a toluene-based glue. Gold contacts are connected by the silver wires and silver paste to the holder (see **Scheme S2** for more details of the device).

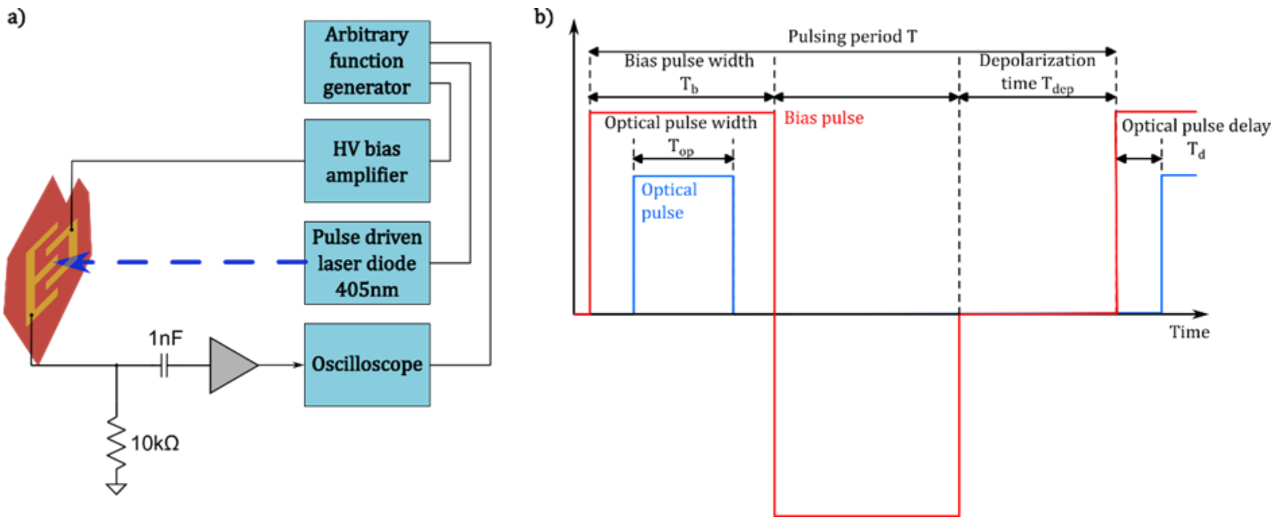

Figure S1. a) Setup for the photocurrent relaxation measurement, **b)** Timing of the optical and bias pulse.

The optical power transients were recorded with a commercial photodetector (Alphalas UPD-200-UP photodiode (τ_r <175 ps) with a time resolution of the oscilloscope: ~250 ps (4 GHz).

Time-resolved microwave photoconductivity (TRMC) and time-resolved photoluminescence (TRPL) measurements

Samples were excited using a picosecond pulsed laser (532 nm) operating at 1 MHz repetition rate and average power density of ~2.0 W/cm^2. Photoluminescence decay at 620 nm was measured using an experimental setup equipped with a monochromator (0.3 m focal length) and a time-correlated single photon counting module (Becker& Hickl SPC-150-NXwith PMC-150-04detector). For TRMC measurement sample was placed at the open end of a WR28 waveguide. A Gunn diode oscillator was used to generate 38.3 GHz microwaves that were directed to the waveguide. The reflected microwave power was detected using Schottky diode.

Hall effect measurements

Hall effect measurements were carried out with a DX-100 Hall effect system on the $HgPSe_3$ sample with a thickness of 0.257 mm (thickness was measured by a micrometer) at room temperature by making four contacts with silver paste in a van der Pauw geometry (**Scheme S3**). During the measurements, a magnetic field of ±100-500 mT was swept.

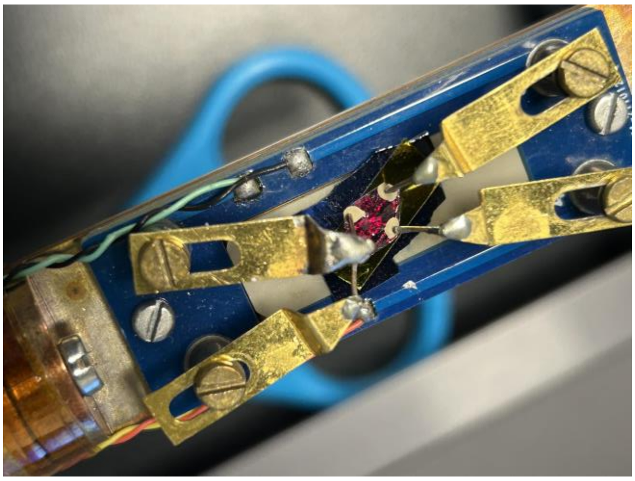 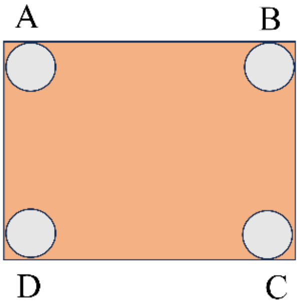

Scheme S3. The setup for the Hall effect measurements and the schematic diagram of the van der Pauw four-point geometry.

The Hall voltage (V_H) is the voltage between two opposing sides of the sample. To calculate the Hall voltage, potential differences were averaged from AC and BD to minimize any errors during measurements.

$V_H = (U_{AC} + U_{BD} + U_{CA} + U_{DB})/4$

The Hall coefficient (R_H) was calculated by the below formula:

$$R_H = \frac{V_H d}{I_A B} \times 10^8$$

where I_A is the current. B is the magnetic field, and d is the thickness of the sample.

The bulk carrier concentration (n) was calculated by the following equation:

$$n = \frac{1}{eR_H}$$

where e is the electron charge (1.60 x 10^{-19} Coulombs)

The sheet carrier density (n_{sheet}) was calculated by the following formula:

$$n_{sheet} = n \times d$$

The mobility (μ) was calculated by:

$$\mu = \frac{1}{ne}\sigma = R_H\sigma$$

The resistivity was calculated by:

$$\rho = \frac{\pi d}{ln2}\frac{RR_{AB,CD+}R_{BC,DA}}{2}$$

And the conductivity:

$$\sigma = \frac{1}{\rho}$$

Below in **Table S2** are the calculated values for different magnetic fields between 100-500 mT:

Table S2. Parameters obtained from Hall data measurements for different magnetic fields.

Magnetic Field (±mT)	Hall Voltage (mV)	Hall Coefficient (cm^3 C^{-1})	Bulk Carrier Concentration (cm^{-3})	Sheet Carrier Concentration (cm^{-2})	Resistivity (Ω^{-1} cm^{-1})	**Mobility (cm^3 V^{-1} s^{-1})**
500	4.36	22434	2.79×10^{14}	2.01×10^{13}	322.85	**69.48**
400	4.22	27156	2.31×10^{14}	5.95×10^{12}	304.07	**89.32**
300	2.12	18175	3.44×10^{14}	8.84×10^{12}	312.44	**58.18**
200	1.34	17257	3.62×10^{14}	9.31×10^{12}	33.535	**52.01**
100	0.60	15456	4.04×10^{14}	1.04×10^{13}	333.27	**46.38**

DFT and calculation of trap density

We first carried out DFT calculations for the $HgPSe_3$ bulk crystals which enabled us to derive the electron (hole) effective mass termed as m_e* (m_h*). The predicted band gap of $HgPSe_3$ was calculated at 1.1 eV. The procedure adopted for extracting electron (hole) effective mass from the DFT-derived conduction (valence) band was based on the effective mass fitting near the gamma (Γ) point. To obtain the electron and hole effective masses, the harmonic E(k) dispersion relation near the Γ was used following the equation,

$E(k) = E_0 \pm \hbar k / 2m_{e,\,h}*$

where, E_0 is the energy eigenvalue of selected conduction minimum (CBM), or valence band maximum (VBM) used for the effective mass. The $HgPSe_3$ bulk crystal's electron and hole effective masses are $0.260m$ and $0.950m$, respectively.

Density of states at C.B (Nc):

$$N_C = 2\left(\frac{2\pi m^* K_B T}{h^2}\right)^{\frac{3}{2}}$$

$$k_\beta = 1.38 \times 10^{-23} J/K$$

$h = 6.626 \times \ 10^{-34} Js$

$$m^* = 0.260m = 0.260 \times 9.10 \times 10^{-34} kg = 2.366 \times 10^{-31} kg$$

where *m* is the mass of the free electron

$$N_C = 2\left(\frac{2 \times 3.14 \times 2.366 \times 10^{-31} \times 1.38 \times 10^{-23} \times 300}{(6.626 \times 10^{-34})^2}\right)^{\frac{3}{2}}$$

$$= 3.31 \times 10^{24} m^{-3} = 3.31 \times 10^{18} cm^{-3}$$

Similarly, $N_v = 2.32 \times 10^{19}$ cm^{-3}

Total theoretical carrier density $N_i = \sqrt{N_c N_v} \times e\frac{-E_g}{2k_bT}$ = 5.06×10^9 cm^{-3}

The measured maximum free carrier density is **4.04 x 10^{14} cm^{-3}**

The thermal carrier velocity, V_T is calculated as follows:

$$V_T = \sqrt{\frac{3K_B T_L}{effective\ mass}} = \sqrt{\frac{3\times300\times1.38\times10^{-23}}{2.366\times10^{-31}}} = 2.29 \text{ x } 10^5 \text{ m s}^{-1} = 2.29\times10^7 \text{ cm s}^{-1}$$

Finally, the trap density was deduced as follows:

Trap density = $\frac{1}{V_T\, \tau_t \sigma_{TR}}$

where σ_{TR} is the trap capture cross section and τ_t is the decay time from TRPL or the non-radiative recombination time which was determined at 0.6 ns.

So, the recombination rate is (R) = $1/_{0.6\ ns}$ = **1.67 x 10^9 s^{-1}**

Trap capture cross section:

$$\sigma_{TR} = \frac{R}{V_T n} = \frac{1.67\times10^{9}\ s^{-1}}{2.29\times10^{7}\times4.04\times10^{14}} = \mathbf{1.80\ x\ 10^{-13}\ cm^{2}}$$

So, the trap density can be determined as:

$$\textbf{Trap density} = \frac{\mathbf{1}}{\mathbf{3.60\times10^{-13}\times1.15\times10^{7}\times0.6\times10^{-9}}} \sim \mathbf{10^{14}\ cm^{-3}}$$

Micro-photoluminescence (PL) and reflectance (R) measurements

Micro-PL measurements were taken employing a 532 nm diode-pumped solid-state laser for excitation (power 0.5 mW) and a 50×, NA = 0.55 microscope objective for collecting the emission from the studied sample. A spectrometer consisting of a 0.55 m focal length grating monochromator and a liquid-nitrogen cooled Si CCD array detector was used to analyze the spectra. For R measurements the sample was illuminated with a white light spectrum from a halogen lamp. The light reflected from the sample was dispersed by a 0.5 m monochromator and detected by a silicon photodiode using a lock-in amplifier. For PL and R measurements at low temperatures, the sample was placed in a closed-cycle cryostat. For R measurements it was an ordinary CCS 350 Janis cryostat and for micro-PL measurements, it was a vibration-damped cryostat from Janis.

Femtosecond transient reflectivity

A Pharos femtosecond laser by Light Conversion based on a diode-pumped Yb:KGW crystal with a fundamental wavelength at 1030 nm, pulse duration of ~ 280 fs and a repetition rate of 2 kHz was used for white light generation. For this, the laser fundamental was focused into a 5 mm thick sapphire crystal, resulting in a broadband pulse with a spectrum ranging between 470 nm and 900 nm, which was used as the probe. The differential reflection signal, defined as $\Delta R/R = (p_{\text{pump on}} - R_{\text{pump off}})/R_{\text{pump off}}$ was recorded using a fast visible camera (Stresing FLCC3001-FFT) coupled to a Princeton Instruments spectrometer as a detector. The pump pulses were provided by a Q-switched Nd:YAG laser (Picolo by InnoLas), emitting pulses with

a width of ~ 1 ns at 1064 nm fundamental wavelength. The second harmonic at 2.33 eV (532 nm) of these pulses is sent onto the sample with a $1/e^2$ diameter of 400 μm and a fluence of 300 μJcm^{-2}. To synchronize the pump and probe pulses, the trigger of the Pharos laser at 2 kHz was sent to a digital delay generator (model DG 645 from Stanford Research Systems) with less than 25 ps rms jitter and used to trigger the Picolo to control the relative delay between pump and probe pulses.

2. $HgPSe_3$ Structural and Morphological Characterization

The structure of $HgPSe_3$ was identified through X-Ray diffraction (XRD) and Raman spectroscopy. The diffraction pattern of the material corresponds to a monoclinic structure with a space group C2/c symmetry (PDF: 00-031-0858) (**Fig. S2a**). The sharp diffraction peaks indicate the high crystallinity of the material. In addition, the Raman spectrum of $HgPSe_3$ (**Fig. S2b**) revealed four main vibrational modes at 149, 190, 211, and 449 cm^{-1}. Not many reports have identified the origin of Raman modes of $HgPSe_3$ to our knowledge. Nevertheless, layered metal selenophosphites exhibit similar Raman modes originating from the P_2Se_6 units with D_{3d} symmetry. [2] Consequently, the Raman spectrum of $HgPSe_3$ is also dominated by the phonon modes of the P_2Se_6 units in the range of 100-250 cm^{-1}, and the peaks at 149 and 168 cm^{-1} are assigned to the E_g out-of-plane vibrations, while the sharp peak at 212 cm^{-1} to the A^1_g in-plane vibration. A peak at 192 cm^{-1} is most likely associated with the Hg-Se vibration [3] and at 449 cm^{-1} another weak signal is attributed to another A^1_g mode of the P_2Se_6 units.

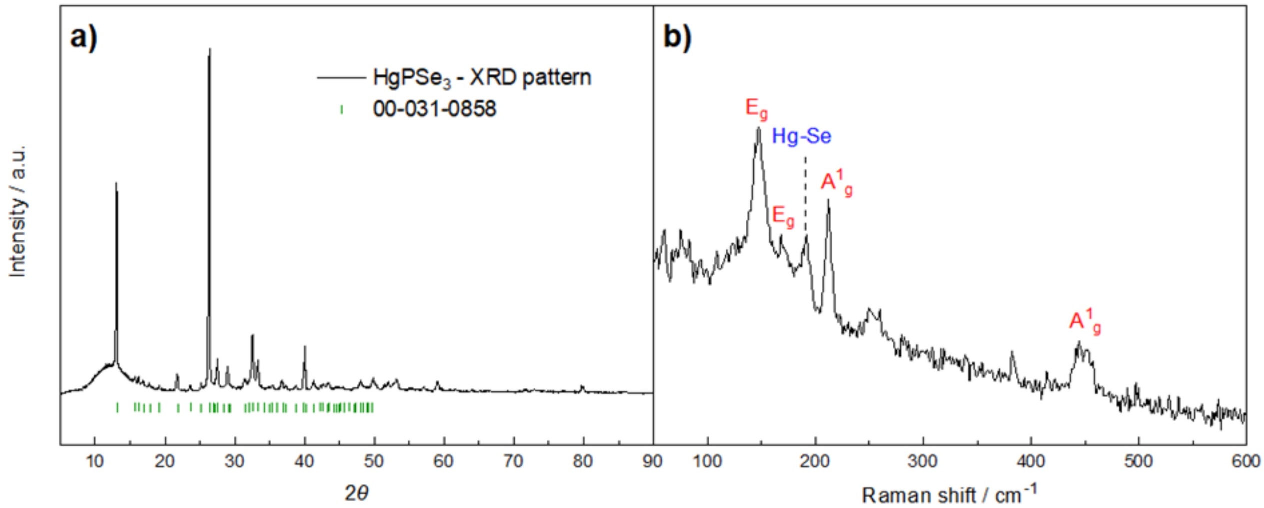

Figure S2. Structural characterization of $HgPSe_3$ **a)** XRD pattern with the corresponding labeled peaks. **b)** Raman spectrum.

The SEM/EDS analysis confirmed the 1:1:3 stoichiometry of $HgPSe_3$ and the uniform distribution of the elements across the material (**Fig. S3**).

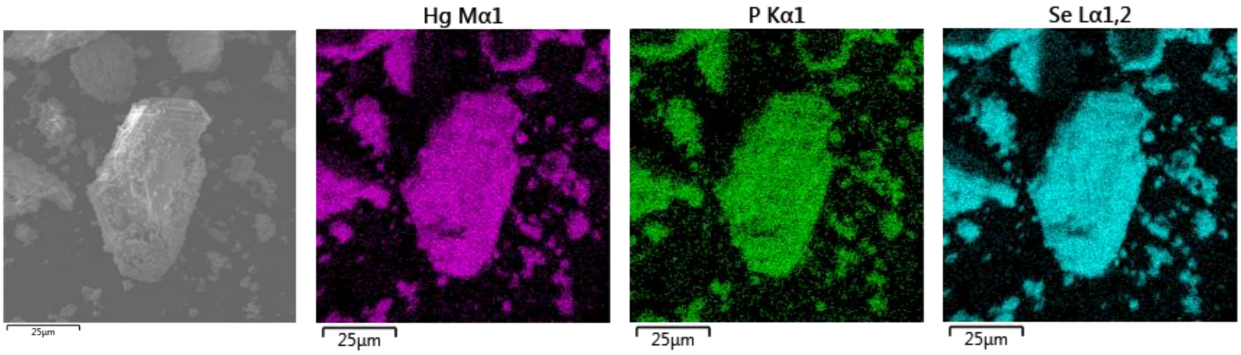

Figure S3. SEM image of $HgPS_3$ with the corresponding EDS elemental maps of mercury, phosphorus, and selenium.

Finally, the $HgPSe_3$ surface composition was investigated through X-ray photoelectron spectroscopy (XPS). The wide-survey spectrum confirmed the presence of mercury, phosphorus, and selenium in addition to adventitious carbon and oxygen due to oxidized species (**Fig. S4a**). Peaks marked with an asterisk are ascribed to the Se LMM Auger transition signals. The high-resolution XPS spectrum of Hg 4f shows two well-defined peaks corresponding to Hg $4f_{7/2}$ and $4f_{5/2}$ with a 4.0 eV spin-orbit separation (**Fig. S4b**). The P 2p

signal was overlapping with the Se LMM Auger transitions creating problems with the deconvolution of the peaks as the Se LMM signal is more intense than the P 2p phosphorus counterpart (**Fig. S4c**). Thus, in addition to the two phosphorus peaks at 133.1 eV (P $2p_{3/2}$) and 134.0 eV (P $2p_{1/2}$) the signal contained another peak due to the Se LMM Auger transition. Lastly, the Se 3d high-resolution XPS peak was deconvoluted into Se $3d_{5/2}$ and Se $3d_{3/2}$ with a spin-orbit of 0.86 eV, ascribed to the Se^{-2} (**Fig. S4d**).

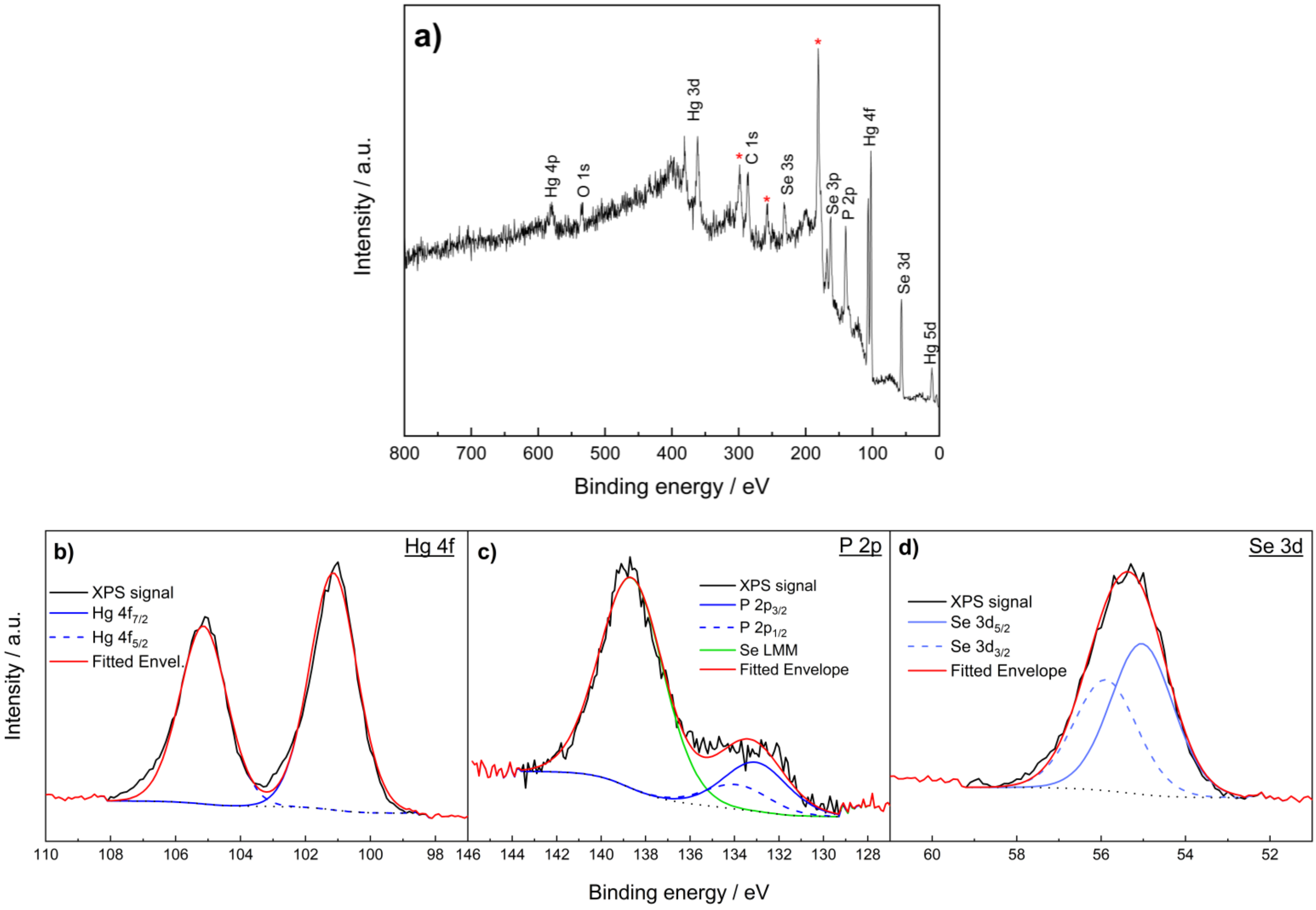

Figure S4. Wide-survey XPS spectrum of $HgPSe_3$. Peaks marked with an asterisk have arisen due to the Se LMM Auger transition. High-resolution XPS spectra of **c)** Hg 4f, **d)** Se 3d, and **e)** P 2p.

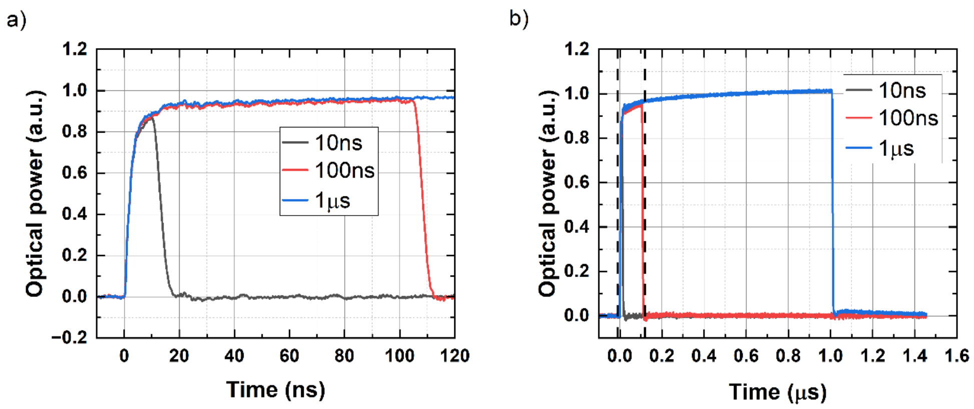

Figure S5. Optical power transient of a 405 nm laser diode for 10 ns, 100 ns, and 1 μs long optical pulses (1 μs width, 1 ms delay, 5 ms + 5 ms bias pulse, 90 ms depolarization). The **a)**-panel is the enlarged depiction of the **b)**-panel in the 0-120 ns region, marked with a dashed frame.

REFERENCES

1. Musiienko, A.; Ceratti, D.R.; Pipek, J.; Brynza, M.; Elhadidy, E.; Belas, E.; Betušiak, M.; Delport, G.; Praus, P. Defects in Hybrid Perovskites: The Secret of Efficient Charge Transport. *Adv. Funct. Mater.,* **2021,** *31,* 2104467
2. Sivadas, N.; Daniels, M.W.; Swendsen R.H.; Okamoto, S.; Xiao, D. *Phys. Rev. B,* **2015,** *91,* 235425
3. Abeykoon, A.M.M., Castro-Colin, M.; Anokhina, E.V.; Iliev, M.N.; Donner, W.; Jacobson, A.J.; Moss, S.C. Synchrotron X-ray and Optical Studies of the Structure of HgSe Semiconductor Nanoclusters Confined in Zeolite L and Zeolite Y. *Phys. Rev. B,* **2008,** *77,* 075333
